# Health and Hell

**Published:** 1889-05

**Authors:** S. H. Preston


### HEALTH AND HELL.

### No. 1.

BY S. H. PRESTON.

Ingersoll says John Calvin was as near like the God of the Old Testament as his health would permit, a remark not meant to be complimentary to either God or Calvin. If good health be akin to godliness, the wicked Colonel would likely hold that the less we had of it, the better, and that it was fortunate for mankind that the Father of Presbyterianism was as infirm as he was.

Now, had Calvin's stomach and physical system been sound and in good order, it is quite probable that his system of theology, so terrible to humane and healthy thinkers, would have been tempered so as to escape their censure. None but a dyspeptic ever dreamed of total depravity, and no one with brain and bowels and liver all right, ever believed in infant damnation. And no reasonably healthy man ever really believed in hell.

The most cowardly and despicable crime that can be committed in our world is to defame a dead man. And no man that ever walked our world has been more maligned and misjudged than John Calvin. That he was a great and gifted and god-fearing man, none of his most virulent villifiers ever ventured to deny. His "Institutes," published at the age of twenty-six, is the most marvelous product of intellect, upon this planet. Take it all in all, together with the circumstances under which it was composed, and the work is not only a prodigy of theologic thought, but an astonishing achievement in the history of literature. It enthroned its author as the intellectual autocrat of his age, and has given a direction to Protestant ideas down to our day.

John Calvin can be considered the Augustine of the sixteenth century, the logician of the reformation ; and his transcendent talents are still to be traced through all the penetrations of Protestant opinions. From his Genevan stronghold for a generation he shook the whole of western Europe. To ridicule, misrepresent, or disparage such a man, seems supremely flippant and spiteful, and those who do it appear as asses braying at a lifeless lion.

Yet no other man has ever been the butt of such bitter obloquy and general execration. His severe piety, his purity of life, his remarkable abilities and unrivaled labors, have always been admitted by his adversaries. Then, what was the matter with him ? It was infirmity of body more than moral badness. The trouble was more in his temperament than in his theology. His personal appearance would make this apparent to a physiologist or physiognomist. His figure was spare and feeble, his chest contracted, his complexion cadaverous, his lips bluish and bloodless, his forehead furrowed, his eyes glassy and restless, his whole aspect that of a sufferer from a multiplicity of maladies. He seemed to be under an incessant nervous strain.

It is said that in youth he never indulged in the pastimes of his associates, but that he shunned them, and censured their frivolities. In later life he showed a singular aversion to society. He slept little and studied much. He became prematurely gray and careworn. Among the list of afflictions from which he was a life-long sufferer were asthma, gout, stone, dyspepsia, and fever.

Calvin was a cold, harsh, unmagnetic man, stern, sour, taciturn, irritable and austere. His demeanor was doubtless greatly due to his diseases, making him an anomalous mixture of morose meekness, morbid morality, tyrannical temperance, cruel charity, and pitiless piety. The Protestant Schlussenberg thus describes his death: "Calvin died of scarlet fever, devoured by vermin and eaten up by an ulcerous abcess, the stench from which drove away every person." (Theol. Cal. t. III. p. 72.)

He was a man hated and feared by his foes, and who had few friends even among his followers. He was incapable of kindling kindly or fraternal feeling. He was an iron-willed, inexorable, intellectual monstrosity, all head and no heart. He was never known to exhibit any emotion except for the misfortunes of others. His own private griefs and distresses were never betrayed by a single tear. At the death of his first and only child he wrote to a friend, formally informing him of the fact and coldly asking him to come and see him, adding as an inducement, that "they could enjoy themselves, and talk together for a half a day."

Still in his stern and rigid way he was a charitable man. He showed sympathy for the persecuted people of the Vandois valleys, and attempted to comfort them with one of his austere exhortations. At another time, he raised a little fund from his Genevan church for the suffering Huguenots. He might have been a softer-hearted and more social man, had he not been so absorbed in theologic speculation. For days together he would tramp along Geneva's lonely lake, utterly oblivious of the most delightful scenery of Switzerland spread around him. His mind was probably pre-occupied with thoughts of the Trinity or perplexed with the problem of pre-destination.

Instead of making Calvin's health a point of comparison between him and the God of the Old Testament, the witty agnostic could with more justice and propriety have asserted that he was as good and generous and humane a man as his health and creed and circumstances would permit. For it is to his bile and his bible and the accepted beliefs of his age, that we can ascribe all that is blameworthy in his life and teaching.

Calvin has been unjustly judged, by the creed that bears his name. And if it oan be shown that he is accountable for that creed, then let his memory be damned to the day of doom. For the concocting of such a creed, should be considered a crime so colossal against the race as never to be condoned by man or God. It is such a chilling, cruel, cursed creed that its accceptance is enough to blast the brains and petrify the hearts of all who truly try to realize its awful import. Such a creed could only have come from the frightful fancies of a fiend in the throes of a theologic delirium tremens. It has swept down the centuries like a sirocco from sulphuric realms, like a tide of eternal torment from the burning abysms of Tartarus. It is enough to turn to tears the innocent joy of childhood, cause men to curse the mothers that brought them into being, and drape in black despair the portal to immortality. It is a faith that would change the facilities of earth into a seething stream of ceaseless sorrow. It is one that would drown the melodies of mirth, with the maledictions of the damned, hush the murmurous glee of humming bees amid the meadow clover, choke the cheery chirp of wren and robin, and forever stop the summer serenade of every silvery flowing fountain.

Now, whatever may have been the faults, or crimes, or shocking atrocities committed by Calvin. his fame should be rescued from the infamy of that frightful creed. With all his asthma and acerbity, his gout and gloominess, his dyspepsia and hypochondria, and though afflicted with every imaginable human ill and having the heart of a devil right from Hades, he had not the capacity, great as was his genius, of getting up such damnable doctrines. Then it may be said that no man had. Well, we will discuss that later, and endeavor to explain whence they came. Our present purpose is to clear Calvin of the charge. And we challenge ecclesiastical scholars to show that he ever originated one religious opinion.

Not one dogma commonly accredited to Calvin, not one of those fearful "five points" imputed to him, not one of the views he so ably advocated, but was advanced by St. Augustine and St. Thomas Aquinas, and other great authorities of the Christian world ages before him. He presented nothing new pertaining to the main matters of dogmatic divinity, such as the trinity, original sin, free will, grace, predestination, &c. Athanasius, Augustine, Anselm, Abelard, and Aquinas, had gone over all the issues involved in these subjects, and all the important points had been almost as sharply and severely formulated as by Calvin himself. There was nearly as great divergence of views respecting these doctrines among Catholic authorities themselves, as there was between Catholics and Protestants. Calvin simply put greater stress upon some, as predestination, and gave them greater theological prominence. But he concocted none of these articles of faith. He simply adopted them as he found them, and advocated them the ablest they had ever been, because they accorded with his dyspeptic condition, and because he devoutly believed the bible from which they were derived. Had he been less bilious he might have believed less and better, and been imbued with brighter hopes of human nature. It is easy for a man with a sour stomach and stone in his bladder, to enjoy an angry God and an awful hell.

Had Ingersoll inherited such a feeble frame as Calvin's he too might have been more like the God of the Old Testament. But with his robust body and jocund health, and happy relations in life, he can endorse the remark of Theodore Parker to a Presbyterian pastor, "your God is my devil." A man with a sound stomach and good digestion, and delightful surroundings in the world, will not be apt to let thoughts of hell destroy his appetite or disturb his sleep. More about Calvin next month.

